# Supplementary material for: Content-rich biological network constructed by mining PubMed abstracts
Source: BMC Bioinformatics. 2004 Oct 8;5:147. doi: 10.1186/1471-2105-5-147 (PMC528731; doi:10.1186/1471-2105-5-147)
Supplement: Additional File 2 — The original results of the above study (non-essential files are deleted to keep the file size under the limit set by BMC bioinformatics). [file 1471-2105-5-147-S2.bz2 › chilibotAdditionalFile2/dip50/208ID8846021E708/html/ITGA1_ITGB1.html]

 


 **ITGA1** and **ITGB1** 
  
Found 245 abstracts in PubMed, retrieved 50.  
 

 What does Google say? 
 PDF only 
| .edu only 

---

**Interactive relationship** (e.g. stimulation, inhibition, etc)

**Inhibitory relationship**- neither alpha CD49d mAb directed against the alpha 4 chain or alpha CD29  [ **ITGB1** ]  directed against the common beta 1 chain of VLA  [ **ITGA1** ]  4 blocked adhesion to BSA or ICAM 1 controls.  Ref: 10854610 J Immunol Methods, 2000
**Neutral relationship**- In addition, the p67 LBP T cells express the integrin beta1  [ **ITGB1** ] , which associates with alpha6 in the laminin specific integrin receptor very late activation Ag VLA  [ **ITGA1** ]  6 alpha6beta1.  Ref: 10477615 J Immunol, 1999

**Non-interactive relationship** (e.g. studied together, co-existance, homology, etc.)

- A significant higher expression of CD29  [ **ITGB1** ]  beta1 integrin p = 0.001, VLA  [ **ITGA1** ]  3 p = 0.002, VLA  [ **ITGA1** ]  4 p = 0.03, and VLA  [ **ITGA1** ]  5 p = 0.001 were observed on PBMCs of chronic alcoholics, compared with control subjects.  Ref: 10069570 Alcohol Clin Exp Res, 1999
- To identify the mechanisms that cause monocyte localization in infarcted myocardium, we studied the impact of ischemia reperfusion injury on the surface expression and function of the monocyte fibronectin FN receptor VLA  [ **ITGA1** ]  5 alpha 5 beta 1 integrin, CD49e CD29  [ **ITGB1** ] .  Ref: 10449434 J Clin Invest, 1999
- However, olopatadine had no effect on IL 5 induced CD49d CD29  [ **ITGB1** ]  VLA  [ **ITGA1** ]  4 expression.  Ref: 12046991 Jpn J Pharmacol, 2002
- Data obtained are presented in concurrence with Clusters of Thymic Epithelial Staining CTES classification for various monoclonal antibodies recognizing CD antigens CD1, CD1a, CD6, CD9, CD14, CD16, CD29  [ **ITGB1** ] , CD30, CD32, CD44, CD45RB, CD47, CD48, CD49a  [ **ITGA1** ] , CD49b, CD49c, CD49d, CD49e, CD49f, CD51, CD53, CD54, CD56, CD57, CD63, CD85, CD95, CD98, CD102, CD103, CD106, CD109, CD146, CD147, CD148, CD151, CD152, CD158a, CD158b, CD164, CD165, CD166 and for monoclonal antibodies 1B1 G7, A4, BD46, BLTZ, HP1C5, IND.64, M72, WU947 whose specifities are not yet defined.  Ref: 12725440 Ann Anat, 2003
- c ILDV NH CH2 5CO had little effect on cell adhesion mediated by two other integrins, VLA  [ **ITGA1** ]  5 alpha5, beta1, CD49e CD29  [ **ITGB1** ]  K562 cell adhesion to fibronectin or Leukocyte Function Associated molecule 1 LFA 1, alphabeta2, CD11a CD18 U937 cell adhesion to Chinese hamster ovary cells transfected with intercellular adhesion molecule 1 at concentrations up to 300 microM.  Ref: 10372817 Br J Pharmacol, 1999
- The data show that in the allergic rabbit model of asthma, VLA  [ **ITGA1** ]  4 CD49d CD29  [ **ITGB1** ]  only, is involved in the acute bronchoconstriction, suggesting an involvement of mast cell degranulation.  Ref: 12877819 Pulm Pharmacol Ther, 2003
- In a retrospective immunohistochemical study based on 27 patients with stage IV follicle center lymphoma FCL the expression of CD44standard CD44s, LFA 1 CD11a, CD18, VLA  [ **ITGA1** ]  4 CD49d, CD29  [ **ITGB1** ]  and ICAM 1 CD54 was analysed on lymphoma cells in bone marrow infiltrates.  Ref: 10374862 Leuk Res, 1999
- Anti CD9, CD26, CD29  [ **ITGB1** ] , CD31, CD36, CD44, CD49a  [ **ITGA1** ] , CD49b, CD49c, CD49d, CD49e, CD49f, CD54, CD55, CD56, CD62E &.  Ref: 10235320 J Pediatr Surg, 1999
- Other molecules expressed by the cultured stromal cells include CD29  [ **ITGB1** ] , CD49a  [ **ITGA1** ] , CD49b, CD49d, CD49f, CD51 61, CD54, CD55, CD56, CD58, CD59, CD61, CD71, CD79b, CD81, CD82, CD90, CD95, CD107a, CD130, and CD147.  Ref: 10951495 Prostate, 2000
- The upregulation of CD29  [ **ITGB1** ]  and VLA  [ **ITGA1** ]  proteins only affected T lymphocytes CD2 CD8 CD4 cells.  Ref: 10069570 Alcohol Clin Exp Res, 1999
- The up regulation of different adhesion molecules such as VLA  [ **ITGA1** ]  4 CD49d CD29  [ **ITGB1** ]  and ICAM 1 CD54 on the pulmonary endothelium and leukocytes, is a key event in sepsis induced lung injury leading to inflammatory tissue alterations.  Ref: 11355415 Int J Legal Med, 2001
- Post mortem markers of sepsis an immunohistochemical study using VLA  [ **ITGA1** ]  4 CD49d CD29  [ **ITGB1** ]  and ICAM 1 CD54 for the detection of sepsis induced lung injury.  Ref: 11355415 Int J Legal Med, 2001
- A significant loss of the VLA  [ **ITGA1** ]  3 CD49c CD29  [ **ITGB1** ]  adhesion receptor was observed whereas all other intergins analyzed lacked considerable changes.  Ref: 10851427 Cancer Biother Radiopharm, 1998
- Many of these cells also express LFA 1 CD11a CD18, Mac 1 CD11b CD18, and VLA  [ **ITGA1** ]  4 CD49d CD29  [ **ITGB1** ]  on their surfaces.  Ref: 11264784 Semin Dial< MedlineTA>Semin Dial, 1998
- In this study, the requirement of cell adhesion molecules, CD11a CD18 LFA 1, CD54 ICAM 1, CD49d CD29  [ **ITGB1** ]  VLA  [ **ITGA1** ]  4, and CD106 VCAM 1, for T cell infiltration into tumors and antitumor function was investigated.  Ref: 10087180 Cell Immunol, 1999
- With the aim of elucidating whether different pathogenic mechanisms are involved in primary progressive multiple sclerosis, we compared membrane expression of the adhesion molecules ICAM 1 CD54, LFA 1alpha CD11a, VLA  [ **ITGA1** ]  4 alpha 4 beta 1 integrin CD49d CD29  [ **ITGB1** ] , L selectin CD62L and ICAM 3 CD50 in peripheral blood and the serum soluble forms ICAM 1, L selectin, VCAM 1 and ICAM 3 in 89 patients 39 with the primary progressive form, 25 with the secondary progressive form and 25 with the relapsing remitting form and 38 healthy controls.  Ref: 10581223 Brain, 1999
- Upregulated expression of VLA  [ **ITGA1** ]  proteins and CD29  [ **ITGB1** ]  in peripheral blood lymphocytes of chronic alcoholics without ethanol related diseases.  Ref: 10069570 Alcohol Clin Exp Res, 1999
- ATG also contained functional antibodies to the beta1 integrin CD49d CD29  [ **ITGB1** ]  VLA  [ **ITGA1** ]  4, the alpha4beta7 integrin, CD50, CD54, and CD102 but not to CD62L.  Ref: 12640305 Transplantation, 2003
- Indeed, TGF beta induced Pyk2 phosphorylation was inhibited by monoclonal antibodies against VLA  [ **ITGA1** ]  4, VLA  [ **ITGA1** ]  5 and CD29  [ **ITGB1** ] .  Ref: 11683954 Immunology, 2001
- We found that T h 1 cells derived from ovalbumin OVA specific TCR transgenic DO11.10 mice showed significantly higher levels of VLA  [ **ITGA1** ]  2 CD49b CD29  [ **ITGB1** ]  expression than T h 2 cells.  Ref: 12750354 Int Immunol, 2003
- Loss of VLA  [ **ITGA1** ]  3 CD49c CD29  [ **ITGB1** ]  expression in two multidrug resistant Burkitt s lymphoma cell lines.  Ref: 10851427 Cancer Biother Radiopharm, 1998
- Ligation of VLA  [ **ITGA1** ]  4 by the soluble chimera fusion product vascular cell adhesion molecule 1 VCAM 1 Fc or by 2 anti CD29  [ **ITGB1** ]  beta 1 chain monoclonal antibodies mAb rapidly induced adhesion of myelomonocytic cells HL60, U937 to human umbilical vein endothelial cells HUVECs.  Ref: 10887112 Blood, 2000
- By contrast, nonstimulated eosinophils adhered to VCAM 1 through the beta 1 integrin VLA  [ **ITGA1** ]  4 CD29  [ **ITGB1** ] .  Ref: 10477614 J Immunol, 1999
- Moreover since CD29  [ **ITGB1** ] , as part of the beta1 integrins very late antigen 4 VLA  [ **ITGA1** ]  4 and VLA  [ **ITGA1** ]  6, is involved in homing and differentiation of haematopoietic progenitor cells, its characterization in pig is critical to study the interaction of porcine adhesion molecules with human ligands in the induction of donor specific tolerance toward porcine antigens, a process extremely desirable to prevent rejection of xenogeneic organs.  Ref: 10972224 Transplantation, 2000
- Resting CK MVEC were positive for CD29  [ **ITGB1** ] , CD31, CD49a  [ **ITGA1** ]  and CD49e, but expressed most of these antigens at a significantly lower density than did CK MVEC.  Ref: 11025404 J Vasc Res< MedlineTA>J Vasc Res, 2000
- The alpha 4 beta 1 very late antigen VLA  [ **ITGA1** ]  4, CD49d CD29  [ **ITGB1** ]  and alpha 5 beta 1 VLA  [ **ITGA1** ]  5, CD49e CD29  [ **ITGB1** ]  integrins mediate beta 2 CD11 CD18 integrin independent neutrophil recruitment to endotoxin induced lung inflammation.  Ref: 11254723 J Immunol, 2001
- Small, N to C terminal cyclized peptides containing the leucyl aspartyl valine LDV motif from fibronectin connecting segment 1 CS 1 have been investigated for their effects on the adhesion of human T lymphoblastic leukaemia cells MOLT 4 to human plasma fibronectin in vitro mediated by the integrin Very Late Antigen VLA  [ **ITGA1** ]  4 alpha4beta1, CD49d CD29  [ **ITGB1** ] .  Ref: 10372817 Br J Pharmacol, 1999
- Surface integrin Mac 1 CD11b CD18 and VLA  [ **ITGA1** ]  4 CD49d CD29  [ **ITGB1** ]  expression on monocytes and the relative number of monocyte platelet complexes after in vitro activation were significantly elevated in patients with restenosis compared with patients without restenosis fluorescence intensities of 1425 76 vs 1195 7 7 7 vs 65 6 and 47 4 vs 29 3% for for Mac 1, VLA  [ **ITGA1** ]  4 and monocyte platelet complexes, respectively.  Ref: 11553019 Clin Exp Pharmacol Physiol, 2001
- Furthermore, eosinophil recruitment and activation appears to be mediated by a combination of VLA  [ **ITGA1** ]  4 CD49d CD29  [ **ITGB1** ]  and LFA 1 CD18 CD11a.  Ref: 12877819 Pulm Pharmacol Ther, 2003
- Flow cytometry was used to evaluate the expression rate of VLA  [ **ITGA1** ]  4 CD49d CD29  [ **ITGB1** ]  and VLA  [ **ITGA1** ]  5 CD49e CD29  [ **ITGB1** ]  and beta1 integrin activation ability in the transfected K562 cells.  Ref: 12844402 Zhongguo Shi Yan Xue Ye Xue Za Zhi, 2003
- Leukocyte function associated antigen LFA 1, alpha L beta 2, and CD11a CD18 and very late antigen VLA  [ **ITGA1** ]  4, alpha 4 beta 1, and CD49d CD29  [ **ITGB1** ]  are members of integrin type CAM that are predominantly involved in leukocyte trafficking and extravasation.  Ref: 11857637 Med Res Rev, 2002
- and 2 eosinophil expression of the adhesion molecules leukocyte function associated antigen 1 LFA 1, CD11a CD18, macrophage antigen 1 Mac 1, CD11b CD18 and very late activation antigen 1 VLA  [ **ITGA1** ]  4, CD49d CD29  [ **ITGB1** ]  was evaluated by specific Mab staining and flow cytometric analysis.  Ref: 11153596 Eur Respir J, 2000
